# Laparoscopic-assisted anorectoplasty for anorectal malformation with rectobulbar fistula: A two-center comparative study with posterior sagittal anorectoplasty

**DOI:** 10.1097/MD.0000000000035825

**Published:** 2023-11-17

**Authors:** Shiru Ye, Wei Feng, Yan Zhou, Anxiao Ming, Minming Chen, Changzhen Yang, Chen Zheng, Ruyue Gao, Mei Diao, Yi Wang, Long Li

**Affiliations:** a Department of Pediatric Surgery, Capital Institute of Pediatrics, Beijing, People’s Republic of China; b Graduate School of Peking Union Medical College, Chinese Academy of Medical Sciences, Beijing, People’s Republic of China; c Research Unit of Minimally Invasive Pediatric Surgery on Diagnosis and Treatment(2021RU015), Chinese Academy of Medical Sciences, Beijing, China; d Department of General & Neonatal Surgery, Children’s Hospital of Chongqing Medical University; National Clinical Research Center for Child Health and Disorders; Ministry of Education Key Laboratory of Child Development and Disorders; Chongqing Key Laboratory of Pediatrics, Chongqing, China.

**Keywords:** anorectal malformation, laparoscopy-assisted anorectoplasty, posterior sagittal anorectoplasty, rectobulbar fistula

## Abstract

Due to the controversy on the feasibility of laparoscopic-assisted anorectoplasty (LAARP) for the treatment of the anorectal malformation (ARM) with rectobulbar fistula (RBF), this study aimed to compare the outcomes of LAARP and posterior sagittal anorectoplasty (PSARP) for ARM with RBF. Demographic data, postoperative complications, and bowel function of RBF patients who underwent LAARP and PSARP at 2 medical centers from 2016–2018 were retrospectively reviewed. Eighty-eight children with RBF were enrolled, including 43 in the LAARP group and 45 in the PSARP group. There were no significant differences in the sacral ratio (*P* = .222) or sacral agenesis (*P* = .374). Thirty-seven and 38 patients in the LAARP and PSARP groups were followed up for a median of 4.14 years. The postoperative complications were comparable between the groups (*P* = .624), with no cases of urethral diverticulum. The urination of all cases was normal and no evidence of cyst formation was found on MCU or MRI during the follow-up period. The incidence of rectal prolapse was similar between the 2 groups (9.3% vs 17.8%, *P* = .247). The groups had equivalent Bowel Function Score (15.29 ± 2.36 vs 15.58 ± 2.88, *P* = .645), but the LAARP group had better voluntary bowel movement (94.6% vs 84.2%, *P* = .148) by Krickenbeck classification. The intermediate-term outcomes of LAARP show that the urethral diverticulum was rare by the intraluminal incision of the fistular and the bowel function was comparable to that of PSARP in ARM with rectobulbar fistula. However, LAARP was associated with smaller perineal wounds.

## 1. Introduction

Anorectal malformation (ARM) is the most common spectrum of gastrointestinal malformation, reported as 2.0 to 2.5 per 10,000 live births, and occurs more frequently in boys. In total, 7.4% of male patients with ARM are born with rectobulbar fistula (RBF).^[1]^ Currently, posterior sagittal anorectoplasty (PSARP) is the mainstream surgical treatment for ARM with RBF, while laparoscopic-assisted anorectoplasty (LAARP) remains controversial because of its high risk of postoperative urethral diverticulum.^[2–5]^ Previous studies have reported complete dissection and resection of the fistula was difficult using LAARP.^[2,6,7]^ However, LAARP has several unique advantages, such as tunnel formation with minimal trauma and better outcomes on anorectal manometry.^[5,8–12]^ In recent years, laparoscopic correction of ARM with RBF has been modified in an attempt to close the fistular precisely, such as intraoperative measurement of the fistular, trans-perineal transection, and intraluminal incision.^[13–15]^ However, the definitive outcomes of LAARP remain controversial owing to inadequate evidence. In particular, there has been a lack of extensive comparative studies evaluating the effectiveness of LAARP and PSARP for ARM with RBF. This study therefore aimed to evaluate the outcomes in RBF patients undergoing LAARP or PSARP during the same period.

## 2. Methods

### 2.1. Patients

**Figure 1.**
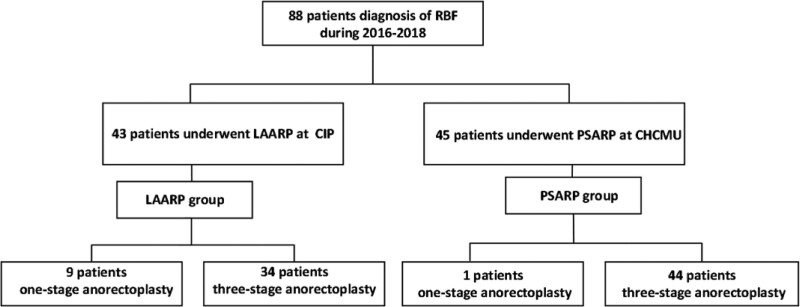
Flowchart of patient enrollment in this study.

Consecutive patients diagnosed with ARM with RBF, who underwent LAARP between January 2016 and December 2018 at the Capital Institute of Pediatrics (CIP) and PSARP at the Children Hospital of Chongqing Medical University (CHCMU), were enrolled in this study (Fig. 1). Patients with previous anorectoplasty and patients with any life-threatening or chromosomal anomalies were excluded. The choice of procedure was not randomized and there was no subjective selection of patients. During the study period, all patients with RBF admitted to CIP received LAARP and those admitted to CHCMU received PSARP. Ethics approval was obtained from the Ethics Committee of the CIP and CHCMU. Written informed consent was obtained from the parents of the patients with ARM before surgery.

### 2.2. Diagnosis

All patients underwent complete medical history, physical, and imaging examinations after admission, including abdominal ultrasonography, echocardiography, prone and cross-table lateral radiography of the pelvis, and pelvic magnetic resonance imaging (MRI). Additionally, all patients received micturating cystourethrography (MCU), and those who underwent staged anorectoplasty also received high-pressure distal colostograms. The diagnostic criteria for ARM with rectobulbar fistula on the high-pressure distal colostogram was the presence of the rectal pouch, located between the pubococcygeal line and the parallel line from the lowest point of the ischial tuberosity, and the fistula orifice located at the bulbo-urethra.^[16]^

### 2.3. Surgical technique

Anorectoplasty was performed by either laparoscopy or with the posterior sagittal approach by 2 surgical teams with experience in > 100 cases of ARM with RBF. The detailed procedures of LAARP,^[8,10,17]^ and PSARP have been previously described in the literature.^[18,19]^ A brief description of the LAARP procedure to manage the fistular is as follow. The distal rectal pouch was dissected along the submucosal layer from 0.5 cm proximal to the urethra. The anterior wall of the distal rectum was incised by a 3-mm cautery hook. The fistular mucosa was peeled off to the junction of the urethral–rectal mucosa. The intraluminal incision enables the surgeon to accurately identify the junction by the laparoscope to secure complete resection of the rectal mucosa. The laparoscope was rotated 180 degrees for better visualization and the muscular cuff was closed with a 5/0 PDS horizontal running suture. The surgical team from CHCMU received systematic training at the Cincinnati Children Hospital Medicdal Center. Anal dilatation was initiated from the second week after anorectoplasty and continued for 3 months. The size of the dilator was gradually increased from a size 8 up to a size 12 in both groups.

### 2.4. Follow-up

All patients were assessed in the outpatient clinic by thorough history taking and physical examination at 1, 3, 6, and 12 months postoperatively, and then every 6 months thereafter. Digital rectal examination was necessary to evaluate sphincter and pelvic floor muscular functions. Pelvic MRI and MCU were routinely undertaken to clarify the presence of the mislocated anal and urethral diverticulum. A colostogram was obtained when a rectal dilatation was suspected. Perioperative data, including age at anorectoplasty, operative time, length of hospital stay, and surgical complications, were evaluated. In terms of the complications, perioperative complications included wound infection, ureteral injury, and ileus within 7 days after the anorectoplasty. Anastomotic complications included perineal wound infections and subcutaneous tissue dehiscence. Rectal prolapse was defined as a protrusion of the rectal mucosa of > 5 mm.^[20]^

In addition to checkup at the regular outpatient clinic between July and November 2021 to evaluate the bowel function, a telephone follow-up of the parents was also conducted. Two resident doctors who had not been involved in the surgical care of patients throughout the potty-training years queried the patients regarding outcomes based on a unified questionnaire to obtain objective results. To evaluate functional outcomes, we used the Krickenbeck classification and the Bowel Function Score (BFS) questionnaire devised by Rintala and Lindah. This classification includes 3 parameters: voluntary bowel movements, soiling, and constipation.^[16]^ Voluntary bowel movements were defined as feeling an urge to defecate, capacity to verbalize this feeling, and ability to hold the bowel movement. Soiling was graded as follows: Grade 1, occasional soiling (up to once or twice per week); Grade 2, soiling every day without social problems; and Grade 3, constant soiling with social problems. Constipation was graded as follows: Grade 1, constipation manageable by changes in diet; Grade 2, constipation requiring laxatives; and Grade 3, constipation resistant to laxatives and diet. The BFS scores were classified as normal (≥ 17), good (12–16), fair (7–15), or poor (≤ 6), in consideration of the ability to hold back defecation, feeling of the urge to defecate, frequency of defecation, soiling, accidents, constipation, and social problems.^[21]^

### 2.5. Statistical analysis

All statistical analyses were performed using SPSS software (version 25.0; Chicago, Illinois, United States). Data were examined for normality. Normally distributed data were presented as the mean ± standard deviation, while data without normal distribution were presented as the median (interquartile range, IQR). Categorical variables were presented as frequencies (percentages). Chi-squared test, independent t-test, and rank-sum test were performed for 2-group comparisons. Statistical significance was set at *P* < .05.

## 3. Results

### 3.1. Patient background data

A total of 88 children with RBF were enrolled in this study, including 43 in the LAARP group and 45 in the PSARP group. All patients completed anorectoplasty successfully, with minimal intraoperative blood loss. Nine patients in the LAARP group and 1 patient in the PSARP group underwent 1-stage anorectoplasty. The rate of patients undergoing colostomy after birth was 97.8% in the PSARP group and 79.1% in the LAARP group (*P* < .01). There was no significant difference in the sacral ratio (0.71 ± 0.07 *vs* 0.68 ± 0.11, *P* = .222) or sacral agenesis (25.6% *vs* 17.8%, *P* = .374) between the 2 groups. The background data and perioperative parameters of the patients are shown in Table 1. The mean age at anorectoplasty was 3.59 ± 2.46 months in the LAARP group and 6.95 ± 3.58 months in the PSARP group (*P<*0.01). The mean body weight at anorectoplasty in the LAARP group was significantly lower than the PSARP group (6.19 ± 1.69 kg *vs* 8.03 ± 1.81 kg, *P<*0.01). The postoperative hospital stay was 9.0 days (IQR, 7.0–11.0) in the LAARP group and 7.0 days (IQR, 7.0–7.0) in the PSARP group (*P* < .01).

**Table 1:** Demographic, associated anomalies, and the clinical features of all patients.

	LAARP (n = 43)	PSARP (n = 45)	*P*
Age at anorectoplasty (mo)	3.59 ± 2.46	6.96 ± 3.68	<.01
Body weight at anorectoplasty (kg)	6.19 ± 1.69	8.03 ± 1.81	<.01
Colostomy			<.01
Transverse colostomy, n (%)	15 (34.9)	44 (97.8)	
Sigmoid colostomy, n (%)	14 (32.6)	0 (0.0)	
Descending colostomy, n (%)	5 (11.6)	0 (0)	
No colostomy, n (%)	9 (20.9)	1 (2.2)	
Sacral ratio	0.71 ± 0.07	0.68 ± 0.11	.222
Sacral agenesis, n (%)	11 (25.6)	8 (17.8)	.374
Operative time (hours)	1.77 ± 0.69	1.59 ± 0.23	.130
Postoperative hospital stays (days)	9.0 (7.0, 11.0)	7.0 (7.0, 7.0)	<.01

LAARP = laparoscopic-assisted anorectoplasty, PSARP = posterior sagittal anorectoplasty.

### 3.2. Complications

The median follow-up period of both the LAARP and PSARP groups was 4.14 years. Overall, there was no significant difference in the incidence of postoperative complications between the 2 groups (14.0 % *vs* 17.8%, *P* = .624) (Table 2). All patients underwent MCU and MRI postoperatively, prior to removal of the Foley catheter removal in the LAARP group and within 7 days after discharge in the PSARP group. And thereafter pelvic MRI, MCU or ultrasonography was carried out every 6 months within 1 year after surgery. We found no evidence of cyst or stone formation on MCU or MRI in any cases. The urination of all cases was normal without daytime dribbling, incontinence, recurrent urinary tract infections, and pain with ejaculation during the period of follow-up. Thus, there were no cases of urethral diverticulum or recurrent fistula in either group.

**Table 2:** Postoperative complications.

	LAARP (n = 43)	PSARP (n = 45)	*P*
Perioperative complications, n (%)	1 (2.3)	0 (0)	.489
wound infection, n (%)	0 (0)	0 (0)	
intestinal obstruction, n (%)	1 (2.3)	0 (0)	
Rectal prolapse, n (%)	4 (9.3)	8 (17.8)	.247
Anastomotic complication, n (%)	0 (0.0)	0 (0.0)	—
Recurrent fistula, n (%)	0 (0.0)	0 (0.0)	—
Urethral diverticulum, n (%)	0 (0)	0 (0)	—
Megarectum, n (%)	1 (2.3)	0 (0)	.489
Overall, n (%)	6 (14.0)	8 (17.8)	.624

LAARP = laparoscopic-assisted anorectoplasty, PSARP = posterior sagittal anorectoplasty.

Six patients in the LAARP group developed complications. Early postoperative intestinal obstruction occurred in 1 patient who received conservative treatment. Rectal prolapse (hemi-circumferential with the protrusion <15 mm) occurred in 4 patients who received surgical repair during colostomy closure. Furthermore, intractable constipation occurred in 1 patient postoperatively, which was confirmed to be megarectum on colostogram. Eight patients developed rectal prolapse in the PSARP group. One underwent surgical repair 2 years after anorectoplasty (circumferential with the protrusion longer than 15 mm), while the remaining 7 cases were hemi-circumferential with a protrusion <15 mm.

### 3.3. Functional outcomes

Thirty-seven patients in the LAARP group and 38 patients in the PSARP group were successfully followed up. The overall response rate was 85.22%. At the time of bowel function assessment, the mean ages between the 2 groups were comparable (4.65 ± 0.90 years *vs* 4.75 ± 0.78 years, *P* = .620). The mean BFS results were similar between the 2 groups (15.29 ± 2.36 *vs* 15.57 ± 2.88, *P* = .645). The distributions of the BFS ranks between the 2 groups were not statistically different (Table 3). Thirty-five (94.5%) patients in the LAARP group and 33 (86.8%) patients in the PSARP group scored good to normal (BFS ≥ 12), while 2 (5.4%) patients in the LAARP group (BFS = 8–11) and 5 (13.2%) patients in the PSARP group (BFS = 7–11) had poor to fair scores.

**Table 3:** Bowel function scores (good to normal: BFS ≥ 16, poor to fair: BFS = 7–15).

n (%)	LAARP (n = 37)	PSARP (n = 38)	
Good to normal	35 (94.5)	33 (86.8)	0.430
Poor to fair	2 (5.4)	5 (13.2)	

BFS = bowel function score, LAARP = laparoscopic-assisted anorectoplasty, PSARP = posterior sagittal anorectoplasty.

**Figure 2.**
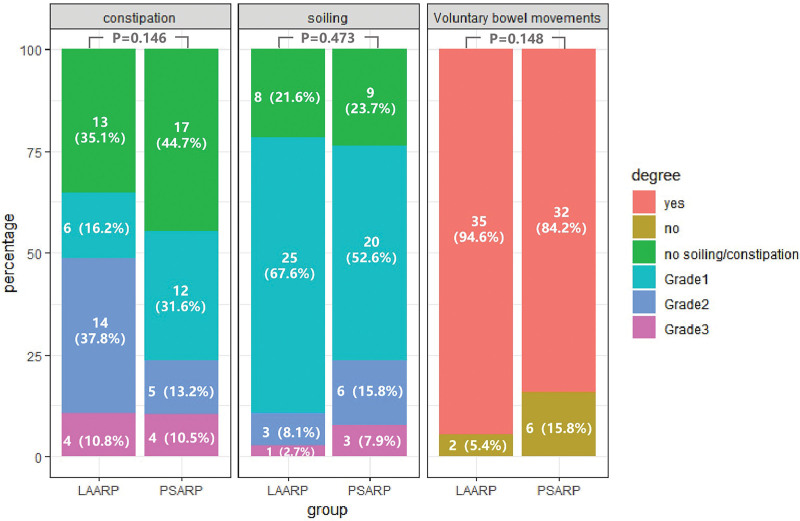
Results of the Krickenbeck classification for ARM with rectobulbar fistula (n = 37 in LAARP group, n = 38 in PSARP group). ARM = anorectal malformation, LAARP = laparoscopic-assisted anorectoplasty, PSARP = posterior sagittal anorectoplasty.

The results of the Krickenbeck classification for the 2 groups were shown in Figure 2. There was no significant difference in the distributions of these scores between the 2 groups. Voluntary bowel movements were present in 35 (94.6%) LAARP and 32 (84.2%) PSARP patients (*P* = .148). Thirty-three (89.2%) LAARP and 29 (76.3%) PSARP patients did not have soiling or Grade I soiling. Thirteen (35.1%) LAARP and 17 (44.7%) PSARP patients had no constipation. Compared to the PSARP group, the LAARP group had a lower incidence of Grade I constipation (16.2% *vs* 31.6%) and a higher incidence of Grade II constipation (37.8% *vs* 13.2%).

## 4. Discussion

There is currently no consensus regarding the role of LAARP in ARM with RBF, for a number of reasons. Firstly, the deep location of the rectal pouch and long common wall of the fistular and rectum increases the difficulty of transabdominal/laparoscopic dissection, leading to a high risk of urethral diverticulum.^[22]^ The incidence of posterior urethral diverticulum appeared to be rare in recent years as distal colostogram was all carried out before repair and surgeons became proficient in PSARP. However, Peña et al reported that 20 cases of RBF developed urethral diverticulum, all of which had previously undergone the transabdominal approach.^[7]^ Alam et al^[2]^ summarized the clinic features of the ARM with RBF who developed urethral diverticulum larger than 5 cm, finding that 25% of those were post-LAARP. Secondly, rectal prolapse is a common complication of LAARP due to the wide mobilization of the rectum. The latest results regarding the long-term outcomes of ARM with RBF from Koga et al reported that the incidence of rectal prolapse was 32% (8/25), which was significantly greater than in the PSARP group.^[22,23]^ Finally, the value of LAARP on bowel function has been questioned, owing to the short follow-up and subjective parameters of the scores.^[22]^

In our study, we found no significant differences in operative time or postoperative complications between the LAARP and PSARP groups. In terms of postoperative complications, no patients in either group experienced urethral diverticulum or recurrent fistula. Notably, there were no cases of urethral diverticulum or injury in the LAARP group, which was attributed to the modified technique, of which the key procedures included: bladder retraction by trans-abdominal suture; opening of the anterior wall of the terminal rectum through limited dissection and intraluminal transection of the fistula (peeling off the mucosal layer from the junction between the fistula and urethra); and rotating the laparoscope 180° for better visualization and closed muscular cuff for those patients with long fistula (>0.5-cm length), the stump of muscular cuff was ligated by Hem-o-Lock clip, and those ≤ 0.5-cm length or fistula located in deeper pelvis, the stump of muscular cuff was sutured by 5-0 PDS.^[10,14,24,25]^ Besides, the median hospital length of stay in the LAARP group was longer than that in the PSARP group (9.0 days vs 7.0 days), which was related to the waiting period of patients in the LAARP group for postoperative MCU before discharge. And those in the PSARP group recieved the postoperative MCU after discharge.

Additionally, the incidence of rectal prolapse was comparable between 2 groups (9.3% vs 17.8%, LAARP vs PSARP), which contradicts the commonly held belief that LAARP may increase the risk of rectal prolapse.^[22,23]^ Indeed, the previously reported incidence of post-LAARP rectal prolapse ranged from 30–59%, which was higher than that of the PSARP control group.^[4,23,26]^ According to clinical experience from CIP, maintaining the correct tension of rectum-anal anastomosis while placing the anoplasty sutures could reduce the rate of rectal prolapse. The modified techniques to achieve this are as follows: (1) to aovid over-extensive mobilization of the rectum, we recommend dissection of the mesorectum from the peritoneal reflection without transection of the superior rectal artery; (2) the redundant rectum and surrounding tissue may be trimmed symmetrically to maintain proper tension of rectum-anal anastomosis (slight invagination of the neo-anus) and avoid the mucosal ectropion of the neo-anus. In support of this, Ming et al reported the incidence of the rectal prolapse 1 years after LAARP was significantly decreased in the modified group (3.7%, 1/27) compared with the conventional group (35.3%, 12/34) for ARM with RBF^[27]^; this provided a practical method to reduce the incidence of rectal prolapse for ARM.

Although the age at anorectoplasty and colostomy types were not comparable between the 2 groups, the sacral agenesis rate and age at follow-up were similar. The sacral agenesis and age at follow-up have been identified as reliable indicators of bowel function in numerous large-scale studies.^[28–31]^ Our results showed that the LAARP group had an equivalent Bowel Function Score (15.29 ± 2.36 *vs* 15.58 ± 2.88, *P* = .645) and better voluntary bowel movement score (94.6% *vs* 84.2%, *P* = .148) on the Krickenbeck classification compared with the PSARP group. The proportion of patients with voluntary bowel movements was higher in the LAARP group than in the PSARP group (94.6% *vs* 84.2%). Intact anal sphincter musculature and proper location in the center of the striated muscle complex (SMC) may be vital determinants of rectal sensation. It also achieved a better cosmesis without wide division of the perineum and SMC. And the incidences of soiling and constipation were comparable between the LAARP and PSARP groups.

This study had several limitations. First, this was a retrospective, 2-center study. The decision related to the opening of a colostomy or operating age were therefore by 2 different surgeons, taking into consideration their experience and the clinical condition of the patients. Additionally, postoperative management was not consistent over the study period in either clinic. A prospective study, which could address these shortcomings, will provide more powerful evidence to support our findings. Furthermore, since both the BFS and Krickenbeck classification are clinical evaluations, bowel function could not be objectively assessed; a supplementary objective evaluation would be more convincing.

## 5. Conclusion

To the best of our knowledge, this is the largest cohort comparison of LAARP and PSARP for the treatment of ARM with RBF. This study showed that LAARP was a safe and effective treatment for RBF without increment of urethral diverticulum by the intraluminal incision of the fistular. The intermediate-term outcomes of LAARP were equivalent to those of PSARP for ARM with rectobulbar fistula. However, LAARP was associated with smaller perineal wounds.

## Author contributions

**Conceptualization:** Long Li.

**Data curation:** Yan Zhou, Shiru Ye, Wei Feng, Minming Chen.

**Formal analysis:** Yan Zhou, Shiru Ye.

**Investigation:** Shiru Ye, Wei Feng, Changzhen Yang, Yi Wang.

**Methodology:** Shiru Ye, Wei Feng, Anxiao Ming, Minming Chen, Changzhen Yang, Yi Wang.

**Project administration:** Anxiao Ming, Mei Diao, Wei Feng.

**Resources:** Long Li, Yi Wang.

**Software:** Yan Zhou, Shiru Ye, Ruyue Gao, Chen Zheng.

**Supervision:** Long Li, Yi Wang, Mei Diao.

**Validation:** Long Li.

**Visualization:** Long Li.

**Writing – original draft:** Shiru Ye, Long Li.

**Writing – review & editing:** Long Li.
